# Co-circulation of multiple arboviruses in acute febrile patients in Yunnan, China, identified by metagenomic sequencing

**DOI:** 10.1128/jcm.01670-25

**Published:** 2026-04-20

**Authors:** Mengyuan Chen, Ying Kang, Min Cheng, Xin Li, Jing Keng, Peng Zhao, Hongtao Sui, Jie Dong, Lilian Sun, Bo Liu, Yongfeng Hu, Jinyong Jiang, Fan Yang

**Affiliations:** 1 NHC Key Laboratory of Systems Biology of Pathogens, National Institute of Pathogen Biology, Chinese Academy of Medical Sciences & Peking Union Medical College 12501, Beijing, China; 2 China Institute of Veterinary Drug Control 620909, Beijing, China; 3 Yunnan Institute of Parasitic Diseases, Kunming, Yunnan, China; Mayo Clinic Minnesota, Rochester, Minnesota, USA

**Keywords:** acute febrile illness, viral metagenomics, chikungunya virus, dengue virus, co-circulation, Bayesian phylogenetics

## Abstract

Arboviruses such as dengue virus (DENV), chikungunya virus (CHIKV), and Zika virus (ZIKV) are transmitted by *Aedes* mosquitoes and mainly circulate in tropical and subtropical regions. With global warming, their geographic range is expanding, increasing their threat to public health. Yunnan Province, China, bordering Southeast Asia, is a hotspot for viral importation due to intensive cross-border mobility. However, systematic surveillance for these arboviruses among acute febrile patients remains insufficient. We performed metagenomic sequencing on serum specimens from 990 acute febrile patients at the China–Myanmar border between 2017 and 2023. The pathogens were confirmed by PCR and viral isolation. Phylogenetic and spatiotemporal analyses were used to infer viral origins and transmission dynamics. In this study, a CHIKV outbreak was confirmed in 2019, with strains closely related to those from Myanmar and Thailand. Four DENV serotypes 1–4 were identified, with the predominant serotype varying annually. ZIKV was detected and closely related to strains from Myanmar. Co-infections were identified, including one case each of CHIKV with DENV-1, CHIKV with DENV-3, CHIKV with ZIKV, and DENV-1 with DENV-2. Bayesian spatiotemporal analysis of CHIKV reconstructed global transmission routes, indicating that the 2019 outbreak in China likely originated in India and spread sequentially through Bangladesh, Thailand, and Myanmar. In addition, we also detected enterovirus, hepatitis virus, Saffold virus, and rhinovirus. This study reveals a comprehensive spectrum of pathogens, including the co-circulation of DENV, CHIKV, and ZIKV, and underscores the potential risk of arbovirus importation into China, highlighting the need for strengthened border surveillance.

**IMPORTANCE:**

Arboviruses, including dengue virus (DENV), chikungunya virus (CHIKV), and Zika virus (ZIKV), are expanding their range and threatening global public health. Yunnan, situated along the China–Southeast Asia border, is highly susceptible to viral introduction. By applying viral metagenomic sequencing to acute febrile patients, this study uncovered a comprehensive spectrum of pathogens and the co-circulation of DENV, CHIKV, and ZIKV. Phylogenetic analyses revealed that arboviruses were closely related to strains from Myanmar and Thailand, indicating possible frequent cross-border viral introductions. Meanwhile, we reconstructed the global transmission pathways of CHIKV through Bayesian spatiotemporal analysis, providing valuable insights for regional prevention and control of arboviruses. These findings demonstrate that Yunnan serves as a critical interface for viral importation and underscore the urgent need to strengthen border surveillance and early warning systems to mitigate the spread of arboviruses.

## INTRODUCTION

Arboviruses pose an important threat to global public health, especially dengue virus (DENV), chikungunya virus (CHIKV), and Zika virus (ZIKV) (1). These viruses are primarily transmitted by *Aedes aegypti* and *Aedes albopictus* and cause illnesses characterized by acute fever, rash, and arthralgia (2). About half of the world’s population is now at risk of dengue, with an estimated 100–400 million infections occurring each year (3). Southeast Asia and South Asia bear the greatest burden, with frequent and prolonged severe epidemics (4). In China, a large dengue outbreak occurred in 2014, affecting more than 40,000 individuals (5). CHIKV is classified into three genotypes: East/Central/South African (ECSA), West African, and Asian. The ECSA genotype gave rise to the Indian Ocean lineage (IOL), which has spread widely across Asia since 2005 (6). To date, CHIKV has been reported in more than 100 countries worldwide (7). However, limitations in diagnostics and co-circulation with DENV often lead to underreporting of CHIKV infections (8). Since 2016, ZIKV has been repeatedly introduced through travelers, and frequent outbreaks in Southeast Asian countries continue to pose a high risk of ZIKV importation and local transmission in China (9). Its association with neurological complications and microcephaly has had a profound impact on human health. Moreover, climate change has expanded the geographic range of many mosquito vectors, while globalization and urbanization have accelerated the spread of infectious diseases (10, 11).

Southeast Asia is one of the world’s highest-risk regions for infectious diseases and a hotspot for the spillover of viral pathogens (12). Ruili and Jinghong, two border cities in Yunnan Province, China, are characterized by frequent trade and population movement with Southeast Asian countries. In these cities, cross-border viral transmission has become a serious challenge in the past decade. The subtropical climate has facilitated the sustained proliferation of *Ae. aegypti* and *Ae. albopictus*, contributing to frequent outbreaks of arboviruses (13). Multiple introductions of DENV and CHIKV into China have been documented (14, 15). Nevertheless, surveillance and research focusing on arboviruses among acute febrile patients in border regions remain insufficient, and little is known about their prevalence and transmission dynamics in this population. Strengthening systematic surveillance of acute febrile patients in high-risk regions is therefore critical.

The highly overlapping symptoms caused by many pathogens increase the difficulty of achieving an accurate clinical diagnosis. Conventional diagnostic methods are largely restricted to targeted molecular or serological assays, which may fail to capture the full spectrum of pathogens and co-infections (16). Viral metagenomic next-generation sequencing (vmNGS), an unbiased and high-throughput approach, enables the comprehensive detection of viruses from clinical specimens (17). This technique overcomes the limitations of traditional diagnostics and allows comprehensive characterization of the virome associated with acute febrile illness (AFI). It is noteworthy that the cost of vmNGS remains relatively high, and both experimental procedures and data analysis require considerable time, making it less suitable for rapid and cost-effective applications. Nevertheless, vmNGS remains highly valuable for multi-pathogen screening and scientific surveillance. In this study, we conducted metagenomic surveillance of acute febrile patients at Yunnan ports between 2017 and 2023. We systematically screened for potential pathogens associated with AFI and performed Bayesian molecular clock phylogenetic analyses to characterize viral evolutionary dynamics. Our findings revealed the co-circulation of multiple arboviruses and provided novel epidemiological insights into their cross-border transmission and regional control.

## MATERIALS AND METHODS

### Specimen collection

**Fig 1:**
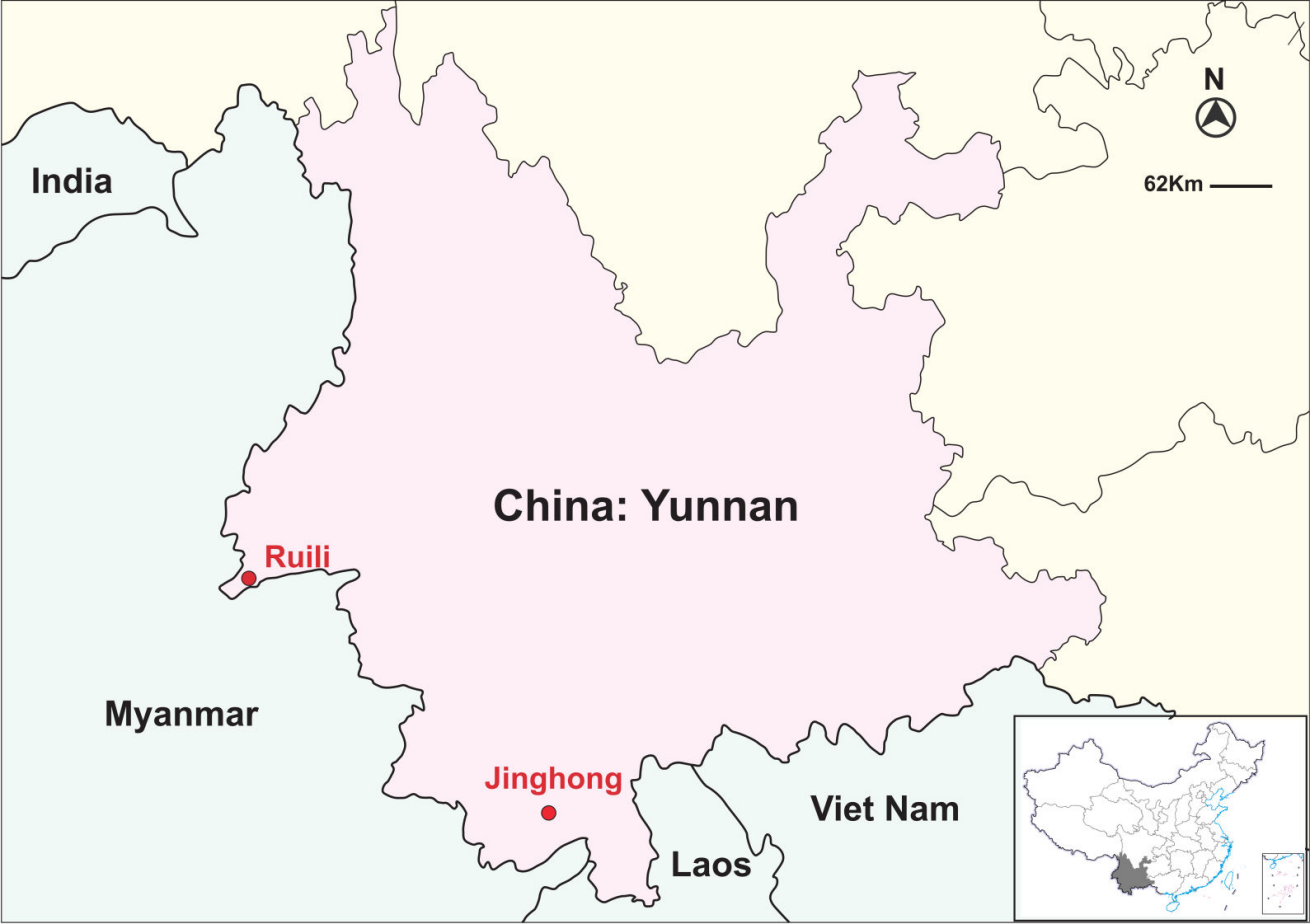
Map showing sampling locations of patients with acute febrile illness in the border regions of Yunnan, China. Red circles indicate sampling sites. Pink shading represents Yunnan Province. Green areas denote neighboring countries bordering southwestern China. Yellow indicates other provinces of China. The inset in the lower right corner shows the location of Yunnan within China (in gray). The map was obtained from the National Platform for Common GeoSpatial Information Services (Tianditu, https://www.tianditu.gov.cn/) and was approved under map review number GS (2019) 3266. The inset map in the lower right corner of Fig. 1 was approved under map review number GS (2020) 4630.

The sampling sites were Ruili and Jinghong, both situated in the border areas of Yunnan Province, China (Fig. 1). A total of 260, 382, and 151 acute febrile serum specimens were collected from Ruili City in 2017, 2018, and 2019, respectively, and 197 specimens were collected from Jinghong City in 2023. All patients were identified and enrolled during routine clinical visits at local healthcare facilities. Owing to the high burden of dengue in these cities, all patients were enrolled according to the following inclusion criteria: acute fever (≥38°C) accompanied by at least one dengue-associated symptom: headache, nausea and vomiting, muscle and joint pain, swollen glands, skin rash, or bleeding manifestations. Serum was isolated from a venous blood draw collected from all dengue-like cases for diagnostic testing. Serum specimens were stored at –80°C until RNA extraction. All specimens were transported under cold-chain conditions and processed promptly to minimize RNA degradation.

### Specimen processing and viral nucleic acid extraction

Individual or pooled specimens (Table S1 for details) were centrifuged at 9,500 × *g* for 10 min. Pooled specimens were used to improve viral detection efficiency and reduce sequencing costs. Equal volumes of each specimen were combined and subjected to viral concentration, ensuring that detection sensitivity was not compromised. The supernatant was filtered through a 0.45 µm polyvinylidene fluoride (PVDF) membrane filter (Millipore, USA) and subsequently concentrated using a 30 kDa ultrafiltration membrane (Millipore, USA) to enrich for viral-like particles. Host nucleic acids were degraded by incubating the enriched specimens with TurboDNase (Thermo Fisher, Germany), Benzonase (Sigma-Aldrich, USA), and RNase (Thermo Fisher, Germany) at 37°C for 30 min in a water bath (18). Viral RNA was then extracted using the QIAamp Viral RNA Mini Kit (Qiagen, Germany). Nuclease-free water was included as a negative control in all experimental procedures.

### Double-stranded cDNA synthesis and PCR amplification

Following RNA extraction, first-strand complementary DNA (cDNA) was synthesized using the SuperScript IV First-Strand Synthesis System (Invitrogen, USA) with 100 pM primer K-8N (5′-GACCATCTAGCGACCTCCACNNNNNNNN-3′). We used the RNA of HeLa cells as a positive control. The single-stranded cDNA was converted to double-stranded cDNA (ds cDNA) using the Klenow fragment (NEB, USA). Random PCR amplification was performed using Premix Taq (Takara, Japan) and primer K (5′-GACCATCTAGCGACCTCCAC-3′). The 50 µL PCR reaction mixture contained 4.7 µL ds cDNA template, 2.5 µL primer K (20 pM), 25 µL Premix Taq, and 17.8 µL nuclease-free water. Thermal cycling conditions were initial denaturation at 94°C for 5 min; 30 cycles of 94°C for 30 s, 50°C for 30 s, and 72°C for 2 min; followed by a final extension at 72°C for 10 min. PCR products were purified using the QIAquick PCR Purification Kit (Qiagen, Germany) and quantified using the Qubit dsDNA High Sensitivity Assay Kit (Invitrogen, USA). Purified products were diluted to 300 pg/µL for library preparation.

### Library construction and sequencing

The libraries were constructed using the Nextera XT DNA Library Prep Kit (Illumina, USA) following the manufacturer’s instructions. Size selection and purification of library fragments were performed using AMPure XP beads (Beckman Coulter, USA). The quality of the constructed libraries was assessed using an Agilent 2100 Bioanalyzer (Agilent, USA). Libraries with a final concentration of ≥3 ng/µL were sequenced on the Illumina NovaSeq PE150 platform at Novogene Biotechnology Co., Ltd. (Beijing, China).

### Genome assembly and annotation

Raw sequencing data were quality-controlled using Trimmomatic v0.39 (19), and human contamination was removed using Bowtie2 v2.5.1 (20). *De novo* assembly was performed with Megahit v1.2.9 (21) to generate contigs, which were subsequently analyzed and annotated using DIAMOND v2.0.15 (22). Taxonomic information was viewed by Megan v6.25.9 (23), and viral sequences were obtained. Genome annotation was performed using Geneious Prime v2024.0.5 (https://www.geneious.com/). The number of reads mapped to viral sequences was counted (Table S1). Genotyping of CHIKV was conducted using the online typing tool available at https://www.genomedetective.com/app/typingtool/chikungunya.

### Confirmatory tests

To minimize the risk of false positive detections and to confirm the number of virus-positive cases within pooled specimens, we validated the metagenomic sequencing results by PCR. RNA was re-extracted from individual specimens to verify the presence of the virus in each patient (Table S1 for details). Specific primers (Table S2) were designed for nested PCR to validate the presence of the sequenced viruses. The first-round amplification was conducted using the One-Step RT-PCR Kit (QIAGEN, Germany), according to the manufacturer’s protocol. The second round of PCR was carried out using Premix Taq (Takara, Japan) with a reaction mixture consisting of 1 μL of the first-round PCR product as the template, 1 μL each of the forward and reverse primers, 25 μL of Premix Taq, and 22 μL of nuclease-free water. The thermal cycling conditions were as follows: initial denaturation at 94°C for 5 min, followed by 35 cycles of denaturation at 94°C for 30 s, annealing at 55°C for 30 s, and extension at 72°C for 1 min, with a final extension at 72°C for 10 min. The target virus was confirmed by visualizing distinct bands on a 2% agarose gel and Sanger sequencing.

### Virus isolation

Clinical specimens containing enteroviruses (EVs) were filtered through a 0.22 µm PVDF membrane filter (Millipore, USA) and inoculated onto human embryonal rhabdomyosarcoma (RD) cells. The cultures were maintained at 37°C in a 5% CO_2_ incubator and subjected to blind passage onto fresh RD cells every 7 days until cytopathic effects (CPE) were observed. When CPE appeared, culture supernatants were collected and clarified by centrifugation. The supernatants were then subjected to PCR amplification, followed by Sanger sequencing to confirm successful isolation of the target virus.

### Sequence identity and phylogenetic analyses

BLASTn searches against the NCBI nucleotide database (https://www.ncbi.nlm.nih.gov/nucleotide) were performed to assess nucleotide identity between the viral sequences obtained in this study and reference genomes (Table S1). Viral nucleotide sequences were aligned using MAFFT v7.520 (24) with default parameters. Maximum-likelihood (ML) phylogenetic trees were inferred using IQ-TREE v2.2.6 (25) with the best-fit substitution model. Branch support was assessed with 1,000 ultrafast bootstrap replicates.

### Evolutionary history analysis

We retrieved all available CHIKV-ECSA sequences (≥9,000 bp) with sampling dates and geographic information from GenBank (https://www.ncbi.nlm.nih.gov/genbank/). Sequences with an N-content exceeding 2% and recombinant sequences were excluded. To simplify the analysis while maintaining temporal and geographic diversity, we excluded sequences with >99.8% similarity that originated from the same country and sampling period. Eleven newly generated CHIKV-ECSA sequences and GenBank sequences with the highest nucleotide identity were included in the data set. Ultimately, a total of 214 sequences comprised the data set for analysis (Table S3). Multiple sequence alignment was performed using MAFFT v7.520, followed by the trimming of low-quality regions at the 5′ and 3′ ends using AliView v1.28 (26). ModelFinder in IQ-TREE 2 identified the GTR+F+I+G4 as the best-fit substitution model, with branch support assessed through 1,000 ultrafast bootstrap replicates. The temporal signal of the data set was assessed using TempEst v1.5.3 (27). Bayesian Markov Chain Monte Carlo (MCMC) sampling was conducted in BEAST v1.10.4 (28). Based on previous molecular clock studies of CHIKV-ECSA, we selected a relaxed uncorrelated log-normal clock model and the Bayesian Skygrid as the Bayesian coalescent tree prior (6, 29). The MCMC analysis was run for 150 million generations, with sampling every 10,000 generations, and was performed independently four times. Computational efficiency was enhanced using BEAGLE. Upon completion, the independent runs were combined using LogCombiner v1.10.4 (30), and a 10% burn-in was removed. Tracer v1.7.2 (31) was employed to verify that all parameters achieved an effective sample size >200. Finally, a maximum clade credibility (MCC) tree was generated using TreeAnnotator v1.10.4 (28) and visualized in FigTree v1.4.4 (http://tree.bio.ed.ac.uk/software/figtree/).

## RESULTS

We performed metagenomic sequencing on serum specimens from acute febrile patients with dengue-like illness. A total of 26 viruses were detected, including arboviruses, viruses associated with enteric and neurological infections, acute hepatitis viruses, blood-borne viruses, and respiratory viruses (Table 1; Table S1).

**TABLE 1:** Viruses detected by vmNGS in dengue-like AFI^*a*^

Virus					Nearest reference accession(s)	Country of nearest reference
	2017	2018	2019	2023		
Arboviruses associated with AFI						
CHIKV			Yes	Yes	MT668625; MN630017, MN974209, MK468801	Myanmar, Thailand
DENV-1		Yes	Yes	Yes	MW793695, MW793710; MW945810, PV344260, PV344274, MW945810; MF405201	Myanmar, Thailand, China
DENV-2			Yes	Yes	MW512476; MZ636805	Singapore, Thailand
DENV-3		Yes	Yes		MW788887	Myanmar
DENV-4	Yes				MW788997	Myanmar
ZIKV		Yes	Yes		MN611472	Myanmar
Virus associated with acute hepatitis						
HEV		Yes			KF176351	China
Blood-borne viruses						
HBV	Yes	Yes		Yes	JQ688405, EU939626, EU305548	China
HCV		Yes	Yes	Yes	OM896920, OM896954, OM896894, MZ161153, KY120328	China
HIV-1	Yes	Yes			AY008717, KF250380	China
Blood-associated viruses						
HPgV		Yes	Yes	Yes	AB021287; PP783773; MW176105; D87713	Myanmar, Denmark, China, Japan
HuBDV		Yes			KY973643	Peru
Respiratory viruses						
HRV-A		Yes			OK017936	USA
HRV-C			Yes		JX025556	USA
Viruses associated with enteric and neurological infections						
CV-A2	Yes				MN419014	China
CV-A4	Yes	Yes		Yes	HQ728260, LC865821; PQ057357, PQ057343	China, Thailand
CV-A6	Yes		Yes	Yes	MG385825, OL840706, OR500230, OR507505, LC791288	China
CV-A10				Yes	OL381909	Viet Nam
CV-A16	Yes				HM459736, EU262658	China
CV-B5	Yes	Yes			JX276378, MN541042	China
EV-A71	Yes				HQ694983	China
ECHO-4	Yes				PP621655, JN203684	Thailand, India
ECHO-6		Yes			MN145871	China
ECHO-9		Yes	Yes		JN203737; AB917074	India, China
ECHO-30	Yes				LC201507	China
SAFV		Yes			MZ384007	Malaysia

^*a*^ CHIKV, chikungunya virus; DENV-1, dengue virus serotype 1; ZIKV, Zika virus; HEV, hepatitis E virus; HBV, hepatitis B virus; HCV, hepatitis C virus; HIV-1, human immunodeficiency virus type 1; HPgV, human pegivirus; HuBDV, human blood-associated dicistrovirus; HRV-A, human rhinovirus A; HRV-C, human rhinovirus C; CV-A2, coxsackievirus A2; EV-A71, enterovirus A71; ECHO-4, echovirus 4; SAFV, Saffold virus.

### Arboviruses associated with AFI

The temporal distribution of six mosquito-borne viruses is summarized (Table 1). Only DENV-4 (*n* = 1) was detected in 2017; DENV-1, DENV-3 (*n* = 1), and ZIKV (*n* = 1) emerged in 2018; DENV-1 (*n* = 2), DENV-2 (*n* = 1), DENV-3 (*n* = 5), ZIKV (*n* = 1), and CHIKV (*n* = 51) co-circulated in 2019; and DENV-1 (*n* = 41), DENV-2 (*n* = 15), and CHIKV (*n* = 3) were again detected in 2023. In 2019, CHIKV circulation was substantial, accounting for 33.8% of AFI cases that year. DENV outbreaks were characterized by annual shifts in the predominant circulating serotype.

Our findings reveal a substantial geographic overlap in the distribution of DENV, CHIKV, and ZIKV, fostering conditions that may facilitate co-infections. We identified three cases of co-infections in 2019: one case of CHIKV and DENV-1 (patient: J25-6), one case of CHIKV and DENV-3 (patient: J29-6), and one case of CHIKV and ZIKV (patient: J31-6). DENV-1 and DENV-2 co-infection was also identified in one case (patient: J11-10) in 2023.

**Fig 2:**
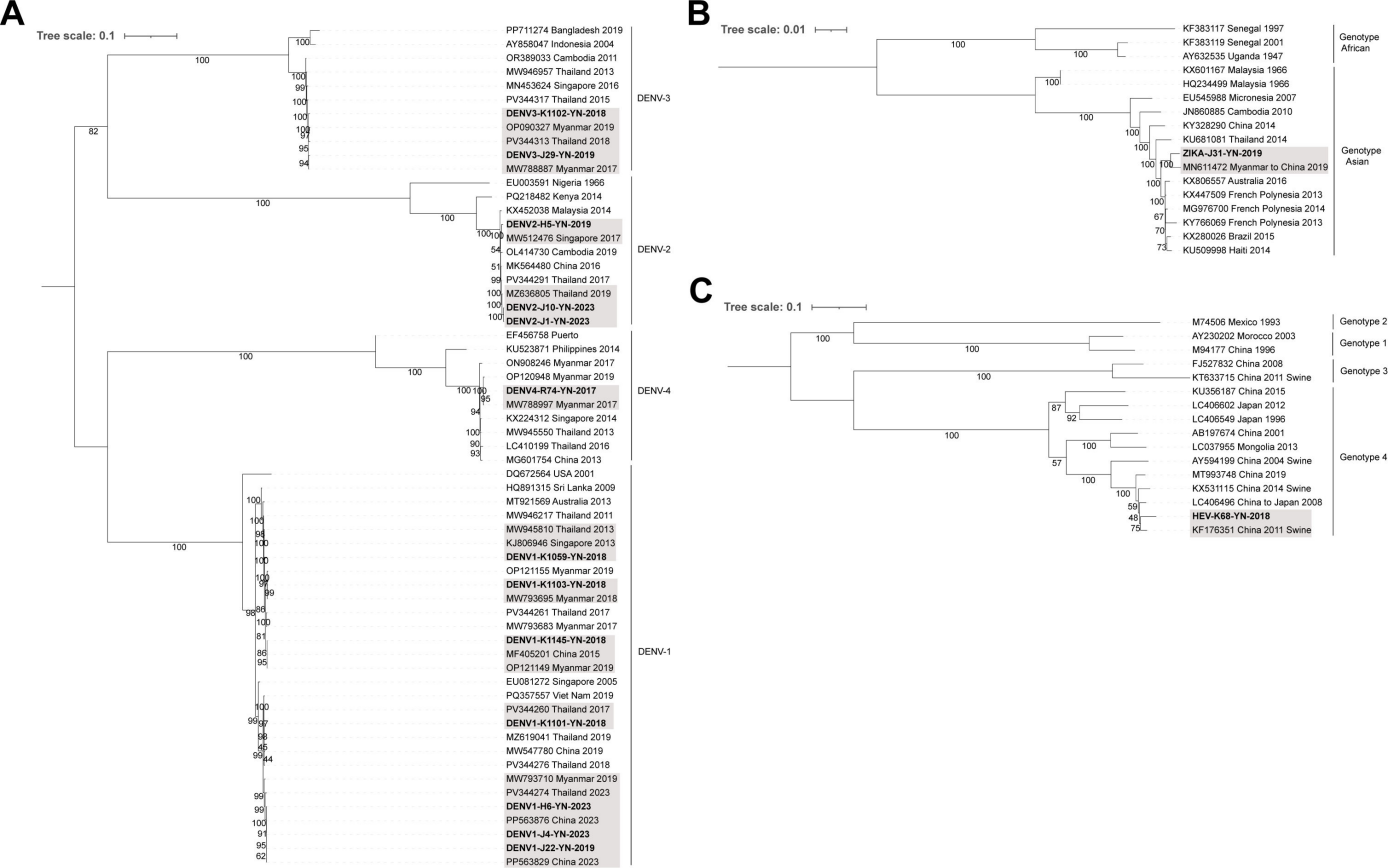
Phylogenetic trees of DENV, ZIKV, and hepatitis E virus (HEV) identified among acute febrile patients in Yunnan Province, China. (**A**) ML tree (*n* = 61) based on complete coding sequence (CDS) of representative DENV. (**B**) ML tree (*n* = 17) based on complete CDS of ZIKV sequences. (**C**) ML tree (*n* = 16) based on partial capsid gene sequences of HEV. Sequences generated in this study are shown in bold. Gray shading indicates reference sequences most closely related to those generated in this study. The scale bars indicate the number of nucleotide substitutions per site. Bootstrap values are shown at the nodes to indicate the statistical support for each branch.

The majority of arboviral sequences exhibited the highest nucleotide identity (over 99%) to strains previously reported from Thailand and Myanmar (Table 1; Table S1). The phylogenetic tree revealed that arboviral sequences detected in Yunnan were interspersed among strains from Thailand, Myanmar, and Singapore, indicating shared evolutionary lineages and possible cross-border transmission (Fig. 2A and B).

### Evolutionary history of CHIKV-ECSA genotype

**Fig 3:**
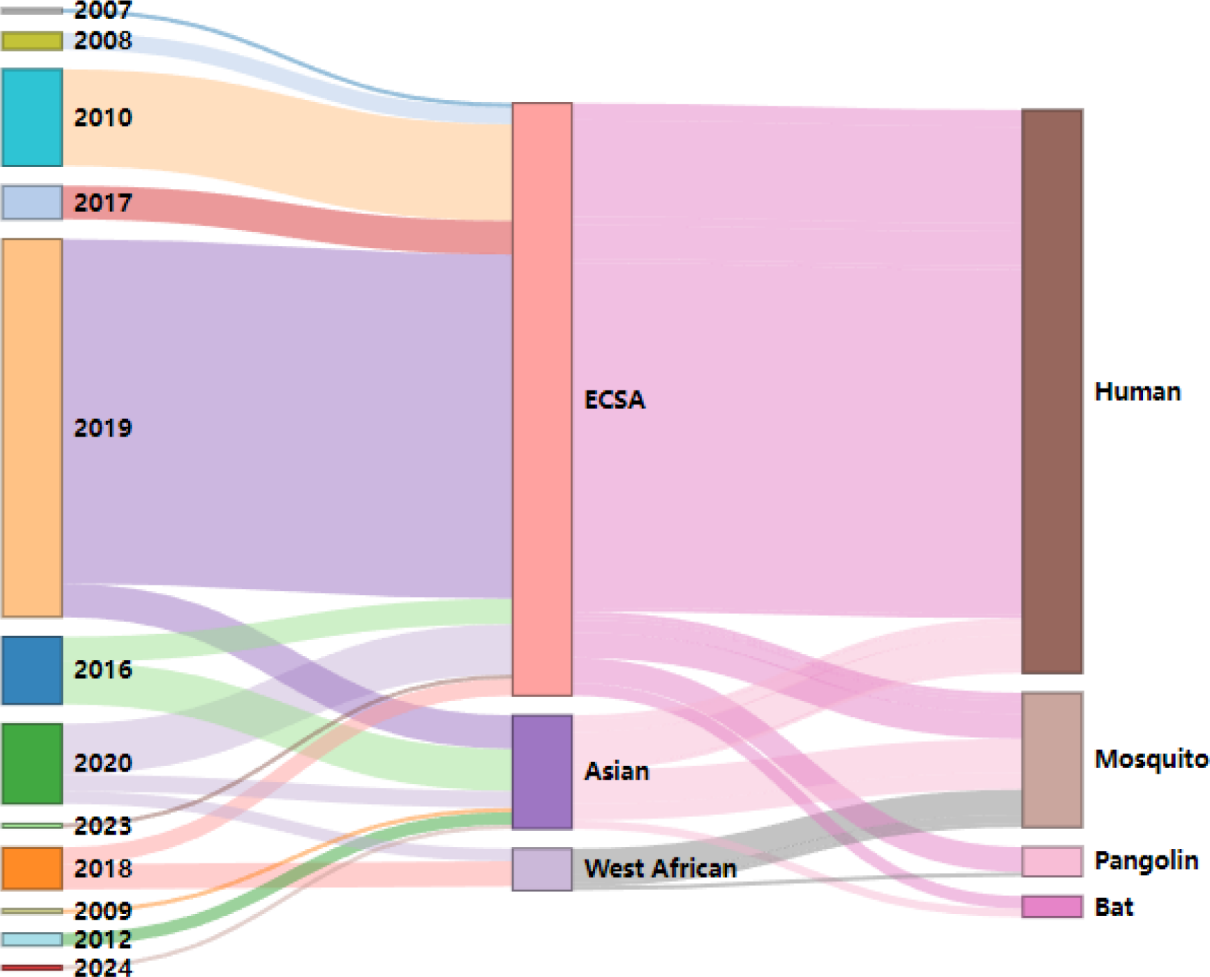
Genotypes and host distribution of CHIKV in China. The figure illustrates the time (left) and host (right) associations of different CHIKV genotypes (middle) detected in China between 2007 and 2024.

We retrieved all available CHIKV sequences from China in GenBank to investigate the genotype and host distribution of CHIKV (Fig. 3). In addition to humans and mosquitoes, CHIKV has also been detected in bats and pangolins. Among human sequences, ECSA genotype was most frequently identified, followed by Asian genotype, with no reported infections of West African genotype in humans in China. Given the widespread detection of the ECSA genotype, we performed molecular clock analysis to explore its evolutionary history.

**Fig 4:**
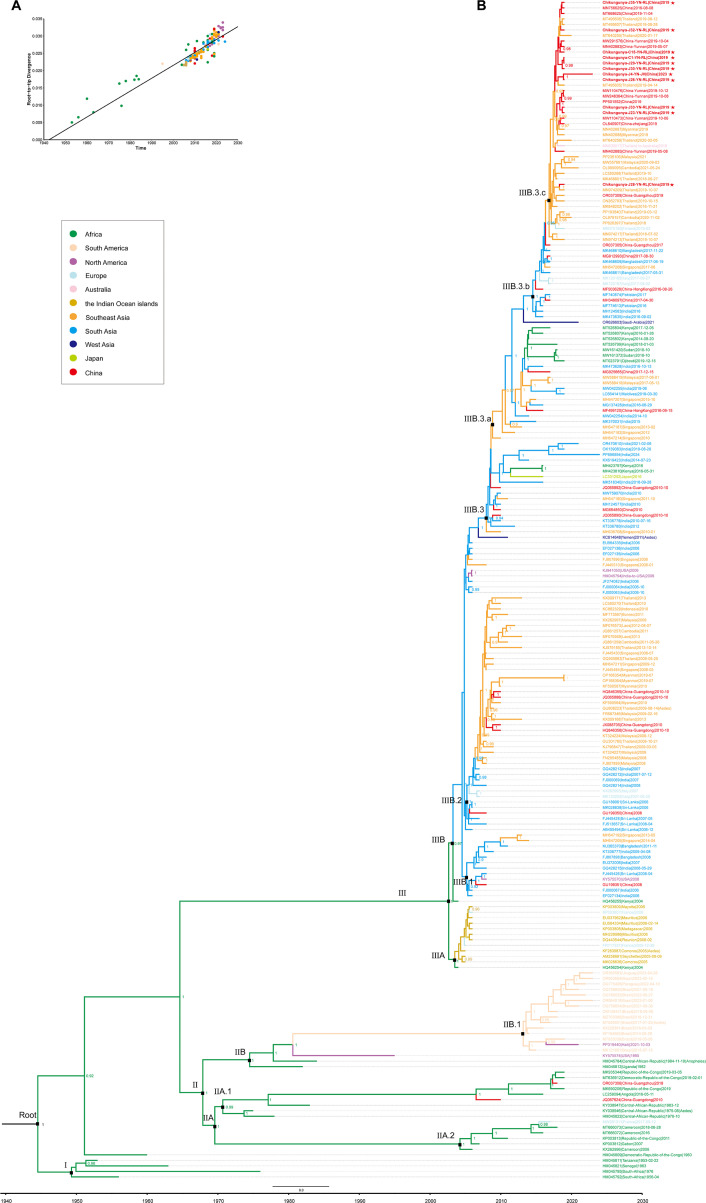
Evolutionary history analysis of the CHIKV ECSA genotype. (**A**) Temporal signal analysis based on root-to-tip regression. Each color represents a different country or region. (**B**) MCC tree of CHIKV-ECSA was inferred from a data set of 214 open reading frame sequences. Sequences obtained in this study are marked with red pentagrams. Branch colors represent the geographic origin of each sequence. Posterior probability values labeled on the MCC tree reflect the level of support for the inferred branching structure, with only those ≥0.9 shown on the tree. The timescale in years is shown on the x-axis. Black squares denote user-defined nodes.

To assess the temporal signal, an initial ML tree was constructed for root-to-tip regression analysis. The estimated time to the most recent common ancestor (tMRCA) was 1,942.39, with an R² value of 0.85 (Fig. 4A). We then reconstructed the MCC tree (Fig. 4B), which revealed three major clades: ECSA-I, ECSA-II, and ECSA-III (ECSA-IOL). The estimated tMRCA for the ECSA genotype was 1,944.54 (95% highest posterior density [HPD]: 1,935.86–1,952.50), with a mean evolutionary rate of 6.71 × 10⁻⁴ (95% HPD: 5.03–7.18 × 10⁻⁴) substitutions per site per year. Key divergence times for each clade are summarized (Table 2).

**TABLE 2:** TMRCA and evolution rates of CHIKV-ECSA lineages^*a*^

Node	Description of the node	PP	tMRCA (95% HPD)	Substitution rate × 10^−4^ (95% HPD)
Root	Root ECSA	1	1,944.54 (1,935.86–1,952.50)	6.71 (5.03–7.18)
I	The origin of ECSA	0.75	1,949.31 (1,945.95–1,952.18)	6.95 (0.53–17.96)
II	ECSA-Africa	1	1,967.88 (1,962.48–1,972.24)	5.09 (0.83–12.17)
IIA	ECSA-Central Africa	1	1,969.16 (1,964.83–1,973.30)	4.97 (0.75–12.04)
IIA.1	ECSA-Central Africa	0.99	1,970.70 (1,966.01–1,973.80)	4.93 (0.70–12.83)
IIA.2	ECSA-Central Africa	1	2,004.23 (2,002.24–2,005.79)	3.64 (2.87–4.50)
IIB	Spread to Americas	1	1,974.49 (1,969.09–1,978.85)	4.57 (1.29–9.63)
IIB.1	ECSA-South America	1	2,013.16 (2,012.12–2,014.00)	3.16 (2.23–4.16)
III	ECSA-Indian Ocean lineage (IOL)	1	2,002.64 (2,001.35–2,003.60)	2.76 (2.05–3.48)
IIIA	IOL-the Indian Ocean islands	1	2,003.50 (2,002.90–2,003.99)	4.24 (0.66–9.80)
IIIB	IOL-Asia	0.97	2,003.24 (2,002.40–2,003.89)	4.34 (0.37–10.83)
IIIB.1	IOL-South Asia	0.71	2,005.19 (2,004.50–2,005.69)	3.07 (0.23–8.21)
IIIB.2	IOL-South Asia to Southeast Asia	1	2,005.21 (2,004.78–2,005.58)	8.73 (1.30–20.58)
IIIB.3	IOL-Asia	1	2,007.95 (2,007.17–2,008.51)	6.56 (1.33–15.54)
IIIB.3.a	IOL-Asia	0.81	2,008.84 (2,008.19–2,009.58)	5.62 (0.75–14.03)
IIIB.3.b	IOL-South Asia to Southeast Asia	1	2,014.52 (2,013.60–2,015.33)	7.73 (1.74–16.31)
IIIB.3.c	IOL-Southeast Asia to China	1	2,016.83 (2,016.42–2,017.32)	11.98 (2.24–25.98)

^*a*^ PP, posterior probability; tMRCA, time to the most recent common ancestor; HPD, highest posterior density; ECSA, East/Central/South African; IOL, Indian Ocean lineage. Units: tMRCA (years); substitution rate (nucleotide substitutions per site per year, ×10^-^⁴).

### Transmission dynamics of CHIKV

**Fig 5:**
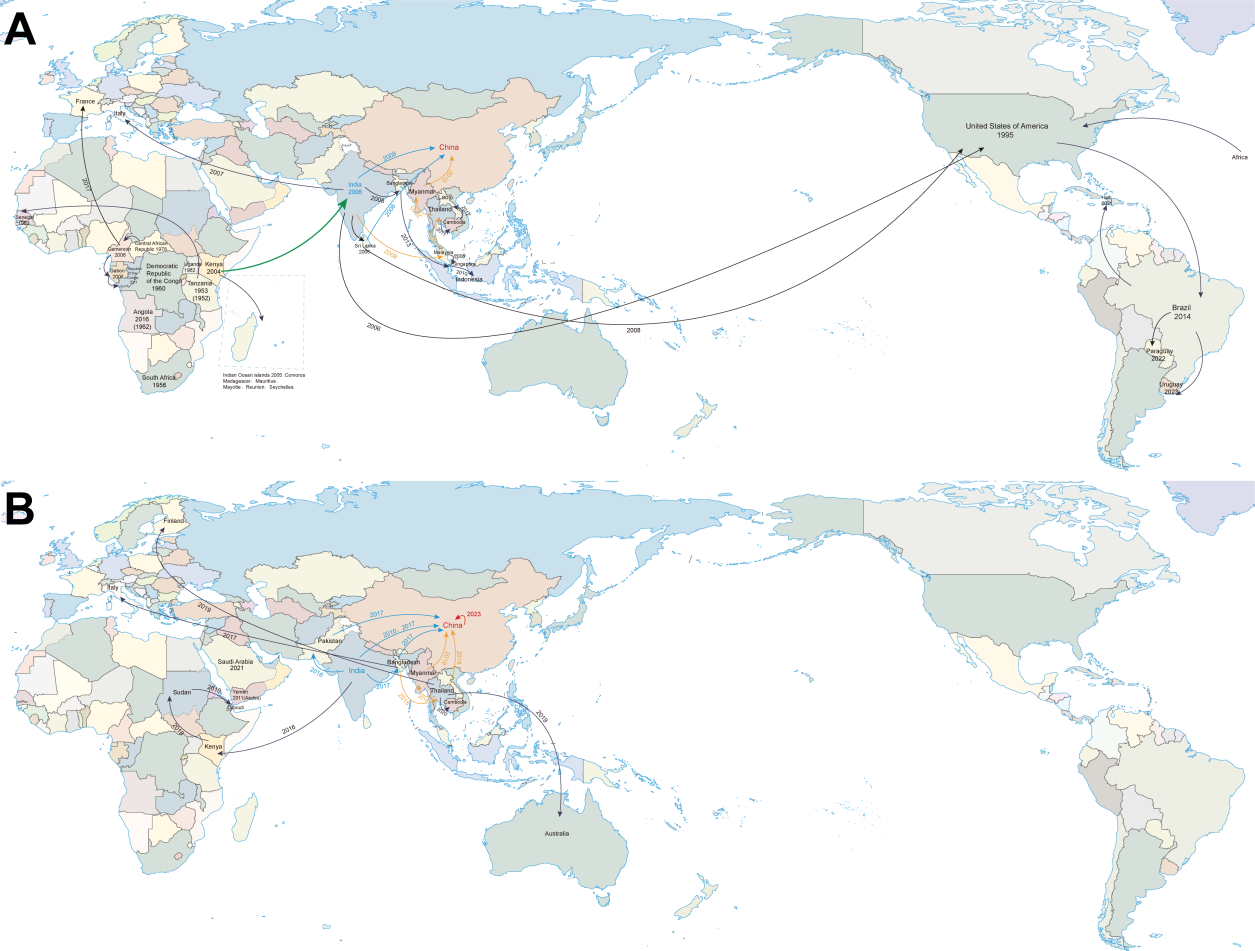
Transmission trajectory map of CHIKV-ECSA. (**A**) This map depicts the transmission routes of CHIKV-ECSA before 2010. (**B**) This map depicts the transmission routes of CHIKV-ECSA after 2010. Time and location are labeled on the map. Green arrows indicate key historical events of CHIKV ECSA genotype entering Asia and the emergence of the IOL lineage. Blue arrows represent routes of introduction from South Asian countries into China. Yellow arrows represent routes of introduction from Southeast Asian countries into China. The map was obtained from the National Platform for Common GeoSpatial Information Services (Tianditu, https://www.tianditu.gov.cn/) and was approved under map review number GS (2016) 1663.

We reconstructed the global transmission pathways of CHIKV-ECSA based on the MCC tree (Fig. 5A and B). The earliest records were reported in Tanzania in 1952 (OR344786) and 1953 (HM045811), followed by its spread to South Africa in 1956 (Fig. 4B: Clade I). During the 1970s to 1980s, CHIKV-ECSA primarily circulated in Central Africa. The first report of CHIKV-ECSA in the USA occurred in 1995, marking its introduction into North America. Around 2,013.16 (95% HPD: 2,012.12–2,014.00), the virus spread from the USA to Brazil, where it has continued to circulate in South America (Clade IIB.1).

The ECSA-IOL lineage (Clade III) emerged around 2,002.64 (95% HPD: 2,001.35–2,003.60), with sub-lineages spreading from Kenya (HQ456254) to the Indian Ocean islands (Clade IIIA) and from Kenya (HQ456255) to India (Clade IIIB), marking the entry of CHIKV-ECSA-IOL into Asia and its establishment in South Asia. CHIKV strains from India and Sri Lanka were likely introduced into China in 2008 (Clade IIIB.1). In the same year, the virus spread from India to Malaysia, triggering outbreaks in Southeast Asia. The 2008 outbreak in Malaysia led to the 2008–2009 epidemic in Thailand, followed by spread to Myanmar and subsequent introduction into China in 2010 (Fig. 5A). Notably, Myanmar strains from 2019 (OP168354 and OP168364) showed high similarity to the 2010 strain (KF590567), suggesting possible local endemic circulation and ongoing risk of reintroduction into China (Clade IIIB.2).

The 2010 outbreak in India also led to CHIKV transmission into China (Clade IIIB.3). A resurgence in India in 2016 likely facilitated further spread to South Asia, Southeast Asia, and China (Clades IIIB.3.a and IIIB.3.b). In 2018, CHIKV was introduced from Bangladesh into Thailand, causing a major outbreak from 2018 to 2019, and subsequently spreading to Myanmar and China (Clade IIIB.3.c). This contributed to the outbreak in Yunnan. Importantly, CHIKV sequences detected in Yunnan in 2023 were highly similar to those from 2019, suggesting low-level endemic circulation of CHIKV in China (Fig. 5B).

### Acute hepatitis virus, blood viruses, and respiratory viruses

One hepatitis E virus (HEV) was identified, and partial capsid gene analysis showed 96.59% nucleotide identity with a swine-origin HEV strain from China (KF176351), classifying it as genotype 4 (Table S1; Fig. 2C). HEV-4 is a well-known zoonotic genotype, primarily transmitted through the consumption of undercooked animal products. Its detection suggests potential foodborne or environmentally linked exposure, although further source tracing is needed. Hepatitis B virus (HBV), hepatitis C virus (HCV), and human immunodeficiency virus type 1 (HIV-1) were frequently detected and showed nucleotide identity with previously reported Chinese strains, indicating ongoing local circulation and highlighting the need for sustained public health interventions and screening efforts. Human pegivirus (HPgV), a blood-associated commensal virus, was detected in 3 years, with reference sequences originating from diverse regions. These findings suggest a global distribution and the potential for long-term asymptomatic persistence in human populations. Human blood-associated dicistrovirus (HuBDV) is an unclassified blood-associated virus. Its partial structural protein gene showed the highest identity (95.23%) to a strain from Peru (KY973643). Additionally, partial VP1 sequences of human rhinovirus (HRV)-A and HRV-C showed the highest nucleotide identity to strains from the USA.

### Viruses associated with enteric and neurological infections

**Fig 6:**
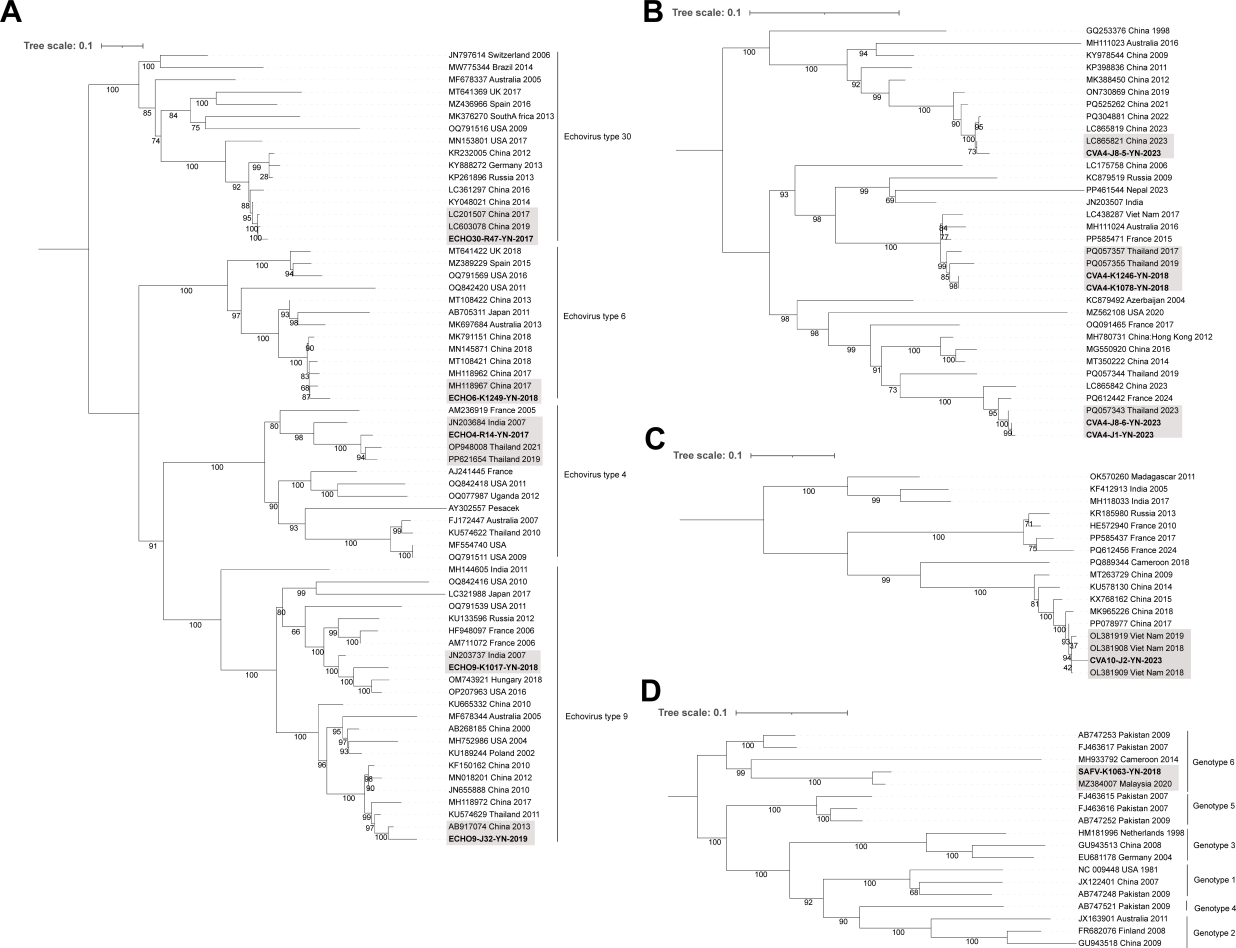
Phylogenetic trees of EV and SAFV identified among acute febrile patients in Yunnan Province, China. (**A**) ML tree (*n* = 65) based on VP1 gene of Echovirus. (**B**) ML tree (*n* = 34) based on VP1 gene of CVA4. (**C**) ML tree (*n* = 17) based on VP1 gene of CVA10. (**D**) ML tree (*n* = 18) based on near-complete CDS of SAFV. Sequences generated in this study are shown in bold. Gray shading indicates reference sequences most closely related to those generated in this study. The scale bars indicate the number of nucleotide substitutions per site. Bootstrap values are shown at the nodes to indicate the statistical support for each branch.

Saffold virus (SAFV) and 11 EVs were detected during surveillance, all of which are recognized etiological agents of hand, foot, and mouth disease, aseptic meningitis, myocarditis, and other neurological disorders. The findings revealed co-circulation of multiple EVs. Coxsackievirus (CV)-A4 and CV-A6 were repeatedly detected across multiple time points, suggesting they may be dominant EV serotypes in the region. Based on VP1 gene sequence analysis, most isolates showed high nucleotide identity to previously reported Chinese strains, indicating potential long-term local circulation (Table 1; Table S1). However, CV-A10 shared the highest nucleotide identity (97.99%) with a Vietnamese strain (OL381909), and phylogenetic analysis showed that it clustered with strains from Vietnam collected in 2018 and 2019 (Table S1; Fig. 6C). SAFV showed 96.46% identity to a Malaysian strain (MZ384007), and both belonged to genotype 6 (Table S1; Fig. 6D). CV-A4, echovirus (ECHO)-4, and ECHO-9 exhibited sequence relationships suggestive of multiple-source introductions or complex transmission chains (Fig. 6A and B). These findings also highlight the potential role of Southeast Asian countries and India in cross-border transmission of EVs into China. ECHO4 (2017) and CVA6 (2023) were successfully isolated in RD cell line (Fig. S1).

## DISCUSSION

Effective infectious disease surveillance in high-risk regions is critical for clinical care and pandemic preemption. vmNGS has become a powerful tool for unbiased pathogen detection (32). Through an optimized metagenomics approach, we conducted continuous surveillance of dengue-like acute febrile patients’ serum specimens in the border regions of Yunnan to infer potential etiologies. The most burdensome and outbreak-prone diseases were arboviruses, including DENV, CHIKV, and ZIKV, among which four cases of co-infection were identified. Preliminary analyses revealed that the strains of arboviruses identified in the border regions of Yunnan exhibited high genetic similarity to Thailand and Myanmar.

DENV has been circulating continuously in the border regions of Yunnan, though the predominant serotype varies annually. CHIKV infection is often characterized by high fever (>38.9°C) (33), and our surveillance identified CHIKV in 33.8% of febrile patients in 2019. However, serological studies indicate that 3%–25% of CHIKV infections are asymptomatic (34). In regions where CHIKV co-circulates with DENV and ZIKV, overlapping symptoms pose significant challenges for clinical diagnosis (35). The actual burden of CHIKV infection could be significantly underestimated. ZIKV was detected in both 2018 and 2019 in our surveillance. The identification of ZIKV is of great importance, particularly among pregnant women and infants. During peak DENV epidemic seasons, pre-existing DENV infections may enhance the pathogenicity of ZIKV, further complicating disease dynamics in the region (36).

During epidemic seasons of arboviruses, co-infections may increase the risk of severe clinical outcomes. In 2023, we identified co-infections of DENV-1 and DENV-2. Globally, approximately 5% of clinical dengue cases progress to dengue hemorrhagic fever (DHF) or dengue shock syndrome (DSS) (37). In settings where multiple DENV serotypes co-circulate, individuals infected with more than one serotype may face an increased risk of developing DHF or DSS (37). In 2019, we also identified CHIKV co-infections with DENV-1, DENV-3, and ZIKV. Co-infection cases have also been reported in Southeast Asian countries, and it has been found that patients with co-infections have an increased risk of DHF (38, 39). Since *Ae. albopictus* and *Ae. aegypti* are capable of transmitting all three viruses, co-infections may emerge as a growing trend.

Following the detection of a CHIKV outbreak in 2019, this study further investigates the transmission dynamics of the ECSA genotype in China. The first isolation of CHIKV in the country dates back to 1986, when it was identified in the brain tissue of a bat from Xishuangbanna, Yunnan Province (40). In 1987, a CHIKV strain was isolated from the blood of an acute febrile patient in the same region (40). The CHIKV outbreak in China occurred in 2019, likely influenced by epidemics in Southeast Asian countries, particularly Thailand and Myanmar. According to Thailand’s national surveillance data, confirmed cases rose sharply from 10 in 2017 to 3,580 in 2018 and 11,721 in 2019 (29). Similarly, CHIKV cases surged in Myanmar during this period (41), posing an increasing risk of importation into Yunnan border regions.

From a historical and global transmission perspective, the Indian subcontinent appears to serve as a central hub for the global spread of CHIKV-ECSA, playing a crucial role in its transmission to Southeast Asia, Africa, Europe, and the Americas. The initial large-scale outbreak of the Asian ECSA-IOL lineage originated in India during 2005–2006, resulting in over 1.3 million CHIKV infections in the country (34). This outbreak subsequently triggered epidemics across Southeast Asian countries before spreading to China. A major outbreak in India in 2016 recapitulated this transmission cycle. In 2019, virus importation from Myanmar and Thailand led to an outbreak observed in China. While sub-Saharan Africa continues to experience sustained CHIKV circulation, its exportation risk remains lower than that of South Asia. In South America, particularly Brazil, CHIKV has caused a major epidemic and has since established sustained local transmission following its introduction in 2013.

In addition to urban transmission between mosquitoes and humans, CHIKV also circulates in sylvatic cycles involving mosquitoes and non-human primates, with potential spillover into urban settings, leading to new outbreak phases (42). For example, although the West African genotype has not been detected in humans in China, its presence in mosquitoes suggests a potential risk of human infection. Although chikungunya fever is a self-limiting disease, it can cause severe long-term sequelae, including persistent joint pain and functional impairment. Perinatal vertical transmission cases have also been reported (15). Given the frequent exchanges between Yunnan border regions and Southeast Asian countries, coupled with global climate change that promotes mosquito proliferation, it is crucial to strengthen international health cooperation and improve viral screening among acute febrile illness. Simultaneously, effective control measures targeting *Ae. albopictus* and *Ae. aegypti* populations should be implemented to prevent the co-circulation of multiple arboviruses.

In addition to arboviruses, EVs have been continuously circulating in the border regions of Yunnan. Eleven EV types were identified over 4 years of surveillance. Co-circulation of diverse EV types may facilitate intertypic recombination. Preliminary sequence analysis suggests both local transmission and possible importation. Therefore, it is necessary to strengthen interregional molecular surveillance and information sharing of viruses, with particular emphasis on the continued monitoring and control of EV-A71 and ECHO that possess neurovirulent potential. SAFV was first reported in 2007 in fecal specimens from febrile children and belongs to the genus *Cardiovirus* within the *Picornaviridae* family (43). It is globally distributed and has been linked to respiratory and gastrointestinal diseases, as well as early-life infections potentially associated with neurological disorders and myocarditis (44). In China, SAFV positivity rate of 1.6%–3.2% has been reported among pediatric patients with enteric or respiratory viral infections (45, 46).

HEV is a known zoonotic pathogen. A study on swine revealed that the HEV RNA positivity rate was 30.2% (47). Swine and swine products are important parts of the food chain and may transmit infectious agents to consumers. The risk of zoonotic transmission of HEV through occupational exposure and consumption of contaminated pork products should not be overlooked. Studies of HPgV have shown that its prevalence ranges from 1% to 5% among healthy blood donors in developed countries, while in developing countries, it can reach up to 20% (48). Dicistroviruses have primarily been described in arthropods (49). In 2018, HuBDV was first identified in the blood specimen of a febrile patient in Peru (50). Subsequently, it was also reported in blood specimens from febrile patients in Tanzania and Nigeria (17, 51). Although its association with clinical disease remains unclear, these findings suggest that human infection with dicistroviruses may be more widespread than previously recognized, warranting further investigation.

This study has several limitations. First, although we employed an optimized viral enrichment strategy, the detection of low-abundance viruses using vmNGS remains challenging, and limitations in clinical specimens availability, genome completeness, as well as the temporal and geographic heterogeneity of the sampled data sets, may have restricted the number of available sequences, thereby limiting our ability to fully capture the genetic diversity of circulating viral populations. Second, the historical evolution and global transmission analysis of CHIKV were based on a subset of available data. Despite our efforts to select a representative and as comprehensive a data set as possible, it may not fully capture the genetic diversity of viral populations, potentially introducing bias into the analysis. Third, we were unable to confirm whether EVs and hepatitis viruses were the true etiological agents of acute febrile illness. Finally, the lack of complete clinical information limited our ability to perform epidemiological and seasonal distribution analyses of the cases.

### Conclusion

We applied metagenomics to perform a comprehensive etiological analysis of acute febrile patients in Yunnan, providing an initial overview of the potential viral pathogen spectrum. Furthermore, we revealed the co-circulation of multiple arboviruses among acute febrile illness in Yunnan and traced the evolutionary history and global transmission dynamics of the CHIKV-ECSA genotype. These findings provide important insights for the control of arboviruses and cross-border health collaboration.

## Data Availability

The sequenced raw reads for this study and the generated genomes have been deposited in NCBI under BioProject PRJNA1240014, PRJNA1240012, and PRJNA1273433 with accession numbers MZ558781, PV797337–PV797355, and PV879479–PV879606.

## SUPPLEMENTAL MATERIAL

Figure S1 jcm.01670-25-s0001.docx Cytopathic effects of CVA6 infection in RD cells compared with uninfected controls.

Table S1 jcm.01670-25-s0002.xlsx Comprehensive information list of all viruses obtained in this study.

Table S2 jcm.01670-25-s0003.xlsx Primer list used in confirmatory tests.

Table S3 jcm.01670-25-s0004.xlsx Metadata of 214 CHIKV-ECSA sequences used in Bayesian phylogenetic analysis.

Table S4 jcm.01670-25-s0005.docx Coverage plots of low-abundance viruses identified by vmNGS.

ASM does not own the copyrights to Supplemental Material that may be linked to, or accessed through, an article. The authors have granted ASM a non-exclusive, world-wide license to publish the Supplemental Material files. Please contact the corresponding author directly for reuse.
