# Supplementary material for: Co-circulation of multiple arboviruses in acute febrile patients in Yunnan, China, identified by metagenomic sequencing
Source: J Clin Microbiol. 2026 Apr 20;64(5):e01670-25. doi: 10.1128/jcm.01670-25 (PMC13170170; doi:10.1128/jcm.01670-25)
Supplement: Figure S1 — Cytopathic effects of CVA6 infection in RD cells compared with uninfected controls. [file jcm.01670-25-s0001.docx]

Figure S1. Cytopathic effects of CVA6 infection in RD cells compared with uninfected controls


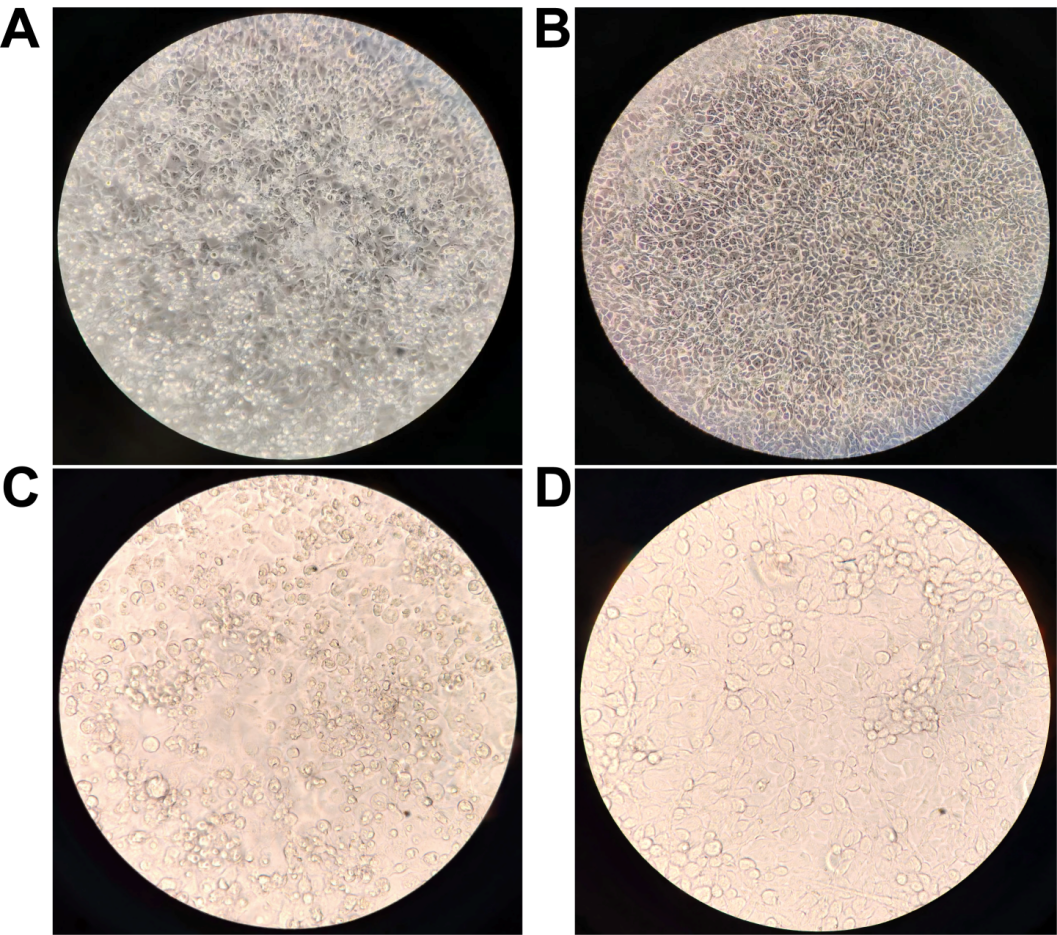


A) RD cells infected with CVA6 at 20× magnification. B) Uninfected RD cells at 20× magnification. C) RD cells infected with CVA6 at 40× magnification. D) Uninfected RD cells at 40× magnification.
