# Supplementary material for: Co-circulation of multiple arboviruses in acute febrile patients in Yunnan, China, identified by metagenomic sequencing
Source: J Clin Microbiol. 2026 Apr 20;64(5):e01670-25. doi: 10.1128/jcm.01670-25 (PMC13170170; doi:10.1128/jcm.01670-25)
Supplement: Table S4 — Coverage plots of low-abundance viruses identified by vmNGS. [file jcm.01670-25-s0005.docx]

Coverage plots of low-abundance viruses identified by vmNGS

| Virus | Year | Coverage plot |
| --- | --- | --- |
| HEV | 2018 | 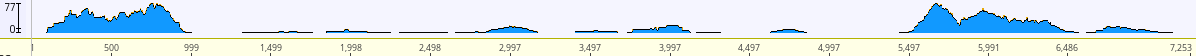 |
| SAFV | 2018 | 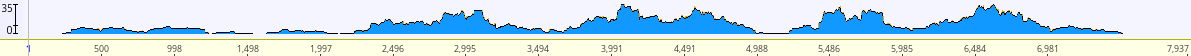 |
| HuBDV | 2018 | 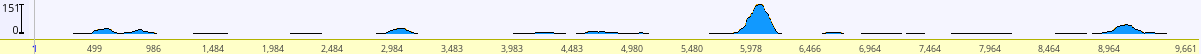 |
| ZIKV | 2018 | 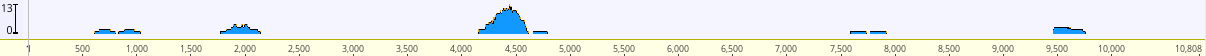 |
| Note: ZIKV: Zika virus; HEV: Hepatitis E virus; HuBDV: Human blood-associated dicistrovirus; SAFV: Saffold virus  The x-axis represents the nucleotide position along the reference genome, and the y-axis indicates the number of reads mapped to each position (coverage). | | |
